# Comprehensive Analysis of Deafness Genes in Families with Autosomal Recessive Nonsyndromic Hearing Loss

**DOI:** 10.1371/journal.pone.0142154

**Published:** 2015-11-11

**Authors:** Tahir Atik, Huseyin Onay, Ayca Aykut, Guney Bademci, Tayfun Kirazli, Mustafa Tekin, Ferda Ozkinay

**Affiliations:** 1 Division of Pediatric Genetics, Department of Pediatrics, School of Medicine, Ege University, Izmir, Turkey; 2 Department of Medical Genetics, School of Medicine, Ege University, Izmir, Turkey; 3 Dr. John T. Macdonald Foundation Department of Human Genetics and John P. Hussman Institute for Human Genomics, Miller School of Medicine, University of Miami, Miami, United States of America; 4 Department of Otolarynghology, School of Medicine, Ege University, Izmir, Turkey; Innsbruck Medical University, AUSTRIA

## Abstract

Comprehensive genetic testing has the potential to become the standard of care for individuals with hearing loss. In this study, we investigated the genetic etiology of autosomal recessive nonsyndromic hearing loss (ARNSHL) in a Turkish cohort including individuals with cochlear implant, who had a pedigree suggestive of an autosomal recessive inheritance. A workflow including prescreening of *GJB2* and a targeted next generation sequencing panel (Illumına TruSight^TM^ Exome) covering 2761 genes that we briefly called as mendelian exome sequencing was used. This panel includes 102 deafness genes and a number of genes causing Mendelian disorders. Using this approach, we identified causative variants in 21 of 29 families. Three different *GJB2* variants were present in seven families. Remaining 14 families had 15 different variants in other known NSHL genes (*MYO7A*, *MYO15A*, *MARVELD2*, *TMIE*, *DFNB31*, *LOXHD1*, *GPSM2*, *TMC1*, *USH1G*, *CDH23)*. Of these variants, eight are novel. Mutation detection rate of our workflow is 72.4%, confirming the usefulness of targeted sequencing approach in NSHL.

## Introduction

Hearing loss (HL) is the most frequent sensory deficit with an incidence of 0.1–0.2% within the newborn population. In developed countries, genetic causes are the most important etiological factors leading to HL. Hereditary hearing loss (HHL) can be syndromic (SHL) (25%) or non-syndromic (NSHL)(75%). While SHL is accompanied by other systemic manifestations, in the non-syndromic form (NSHL), there are no additional findings [1, 2]. Depending on the age of onset and severity of HL, HHL is classified as congenital, prelingual, and postlingual, and mild to profound, respectively.

The genetic transmission of NSHL is autosomal recessive in 75–85% of all cases (ARNSHL), and autosomal dominant in 15–25% of cases (ADNSHL). Small proportion of cases show X-linked or mitochondrial inheritance (1–2%)[3].

Considering the complexity of hearing, many genes play roles in hearing function. It is estimated that, about 1% of human genes (200 to 250 genes) are involved in HHL [4, 5]. To date, more than 80 genes, with more than 1000 mutations, and 140 loci have been identified to be associated with NSHL (http://hereditaryhearingloss.org/). With the exception of few mutations detected recurrently in several genes, such as *GJB2* (MIM 121011) and *SLC26A4* (MIM 605646), most deafness mutations are extremely rare and only seen in either a single or a very few families [6]. Additionally, more than 400 syndromes have been described in the OMIM (Online Mendelian Inheritance in Man^®^) as relating to HL. In some cases, differential diagnosis of SHL and NSHL is very difficult, or even not possible. Furthermore, it has been shown that, both SHL and NSHL can be caused by the mutations in the same gene. This overwhelming genetic and clinical heterogeneity makes the identification of genetic etiology challenging, time-consuming and expensive.

Elucidation of the genetic basis of an individual’s deafness might assist in early diagnosis, developing prevention and/or treatment regimens as well as offering improved genetic counseling and future perspective to probands [3]. With the improvement of laboratory approaches in the genomics era, over the last decade, next generation technologies, such as whole genome sequencing (WGS), whole exome sequencing (WES) or targeted next generation sequencing (TNGS), have assumed revolutionary roles in both research or diagnostic areas.

There have been a number of TNGS panels which included known deafness genes. It is not possible to utilize this tool for the discovery of novel NSHL genes or the genes responsible for other rare diseases. Because of this, we used a targeted enrichment approach for genes known to cause a number of Mendelian disorders (Mendelian exome sequencing (MES)). This strategy consisted of 2761 genes including 63 known NSHL and 39 SHL genes.(S1 Table)

The goal of this study was to identify the genetic etiology of deafness in a group of individuals with NSHL using this MES.

## Materials and Methods

### Patients

Twenty-nine probands who received cochlear implant operation due to congenital or prelingual-onset severe to profound sensorineural HL were included in the study. At least a three-generation pedigree was drawn for each proband. All probands were either born to consanguineous parents or had affected siblings or cousins, suggesting autosomal recessive inheritance. Sensorineural hearing loss was diagnosed with standard audiometric evaluations that included pure tone audiometry, brainstem evoked response, and otoacoustic emissions. No patient exhibited syndromic features during clinical evaluations that included a thorough systemic examination, fundoscopy, EKG, and urinalysis. Written informed consents were provided by all participants. This study was approved by the Ege University Ethics Committee.

### Molecular analysis of *GJB2*

All probands included in the study were screened for mutations in *GJB2*. Genomic DNA was extracted from peripheral leukocytes according to standard protocols. Sanger sequencing for the *GJB2* gene was performed. Each detected variant was then analyzed for cosegregation with deafness in the parents and available family members. A touchdown protocol was used to amplify the targeted DNA region. The amplicons were cleaned up with Sephadex (GE Healthcare). ABI PRISM 3730 DNA analyzer (Applied Biosystems) and Big Dye Terminator Cycle Sequencing V3.1 Ready Reaction Kit (Life Technologies) were used to elucidate the DNA sequence. Variants were named according to NM_004004.5

### Targeted Next Generation Sequencing

TruSight^TM^ Exome focuses on a subset of the exome targeting genes with disease causing mutations which have been shown to be important in specific inherited conditions. This commercially available MES was used in the patients with no mutation in *GJB2*. The coding regions of 2761 genes including total of 63 NSHL genes and 39 SHL genes are covered by the TruSight^TM^ Exome panel (S1 Table). In this gene list, 33 genes have been known to cause purely autosomal recessive nonsyndromic hearing loss (ARNSHL), 19 genes autosomal dominant nonsyndromic hearing loss (ADNSHL), 3 genes X-linked nonsyndromic hearing loss (XNSHL), and 8 genes have been described as being responsible for both ARNSHL and ADNSHL.

Illumina MiSeq platform was used to perform NGS. Sequencing data was analyzed using Illumina VariantStudio variant analysis software and IGV (Integrative Genomics Viewer)[7].

### Data analysis

All genes in this panel were annotated. Firstly, 41 ARNSHL causing genes were evaluated. The homozygous or compound heterozygous variants in ARNSHL causing genes with a frequency of less than 0.5% in public databases (e.g. NCBI dbSNP build141 (http://www.ncbi.nlm.nih.gov/SNP/), 1000 Genomes Project (http://www.1000genomes.org/), Exome Aggregation Consortium (ExAC) (http://exac.broadinstitute.org/) and NHLBI Exome Sequencing Project (ESP) Exome Variant Server (http://evs.gs.washington.edu/EVS/)) were selected [8]. The impact of the mutations on the protein structure was identified using several *in silico* prediction tools such as MutationTaster [9], Polyphen-2 [10], and SIFT [11]. Conservation of residues across species was evaluated by PhyloP algorithm [12] and GERP [13]. Variants identified were categorized according to the ACMG recommendations [14].

Secondly, individuals who had no causative variation in ARNSHL genes were evaluated for known ADNSHL, XNSHL and SHL genes in the MES panel used in this study.

In the third step, patients who had no mutation in the genes related to hearing loss were analysed for all other genes included in the MES panel.

### Confirmation and Segregation analysis

The most likely disease-causing variants, identified by data analysis, were confirmed using direct Sanger sequencing on ABI PRISM 3730 DNA analyzer (Applied Biosystems) and Big Dye Terminator Cycle Sequencing V3.1 Ready Reaction Kit (Life Technologies) and segregation analysis was then performed.

## Results

### *GJB2* mutations

With the screening of *GJB2*, homozygous or compound heterozygous causative mutations were found in 7 probands(24.13%) and all had already been previously reported. Mutation spectrum of *GJB2* is shown in Table 1.

**Table 1:** Causative variants identified in probands.

Family ID	Consanguinity	Gene	Genotype
**Cx1**	No	*GJB2*	c.[35delG];[35delG]
**Cx2**	Yes	*GJB2*	c.[299_300delAT];[299_300delAT]
**Cx3**	Yes	*GJB2*	c.[35delG];[35delG]
**Cx4**	No	*GJB2*	c.[35delG];[333_334delAA]
**Cx5**	No	*GJB2*	c.[35delG];[35delG]
**Cx6**	Yes	*GJB2*	c.[35delG;[35delG]
**Cx7**	Yes	*GJB2*	c.[35delG];[35delG]
**T21**	Yes	*MYO7A*	c.[487G>A]; [487G>A]
**T16**	Yes	*MYO7A*	c.[735G>A];[735G>A]
**T2**	Yes	*MYO7A*	c.[6337G>C];[6337G>C]
**T10**	Yes	*MYO7A*	c.[6487G>A];[6487G>A]
**T14**	No	*TMC1*	c.[534A>C]; [534A>C]
**T15**	Yes	*TMC1*	c.[2050G>A]; [2050G>A]
**T3**	No	*MYO15A*	c.[4642G>A]; [5212-2A>G]
**T4**	Yes	*MARVELD2*	c.[1331+2T>C];[1331+2T>C]
**T6**	Yes	*TMIE*	c.[250C>T]; [250C>T]
**T7**	Yes	*DFNB31*	c.[302C>T]; [302C>T]
**T11**	Yes	*LOXHD1*	c.[71delT]; [71delT]
**T12**	Yes	*GPSM2*	c.[832C>T]; [832C>T]
**T17**	Yes	*USH1G*	c.[355T>C]; [355T>C]
**T19**	Yes	*CDH23*	c.[5545C>G]; [5545C>G]

### TNGS data

On average, 22 probands had 98.70%, 93.33% and 87.17% of mappable bases represented by a coverage of at least 1X, 10X and 20X reads, respectively. Considering only the 41 ARNSHL genes included in TruSight^TM^ Exome the percentage of mappable bases representing by a coverage of at least 1X, 10X and 20X reads were 99.80%, 99.60% and 98.90%, respectively. An average depth of 140.89 reads was achieved. The average numbers of single nucleotide variations (SNVs) and insertions or deletions (INDELs) were 3497 and 64.54, respectively. 93.99% of SNVs and 60.01% of INDELs were found in dbSNP.

### Mutations in known deafness genes

**Fig 1:**
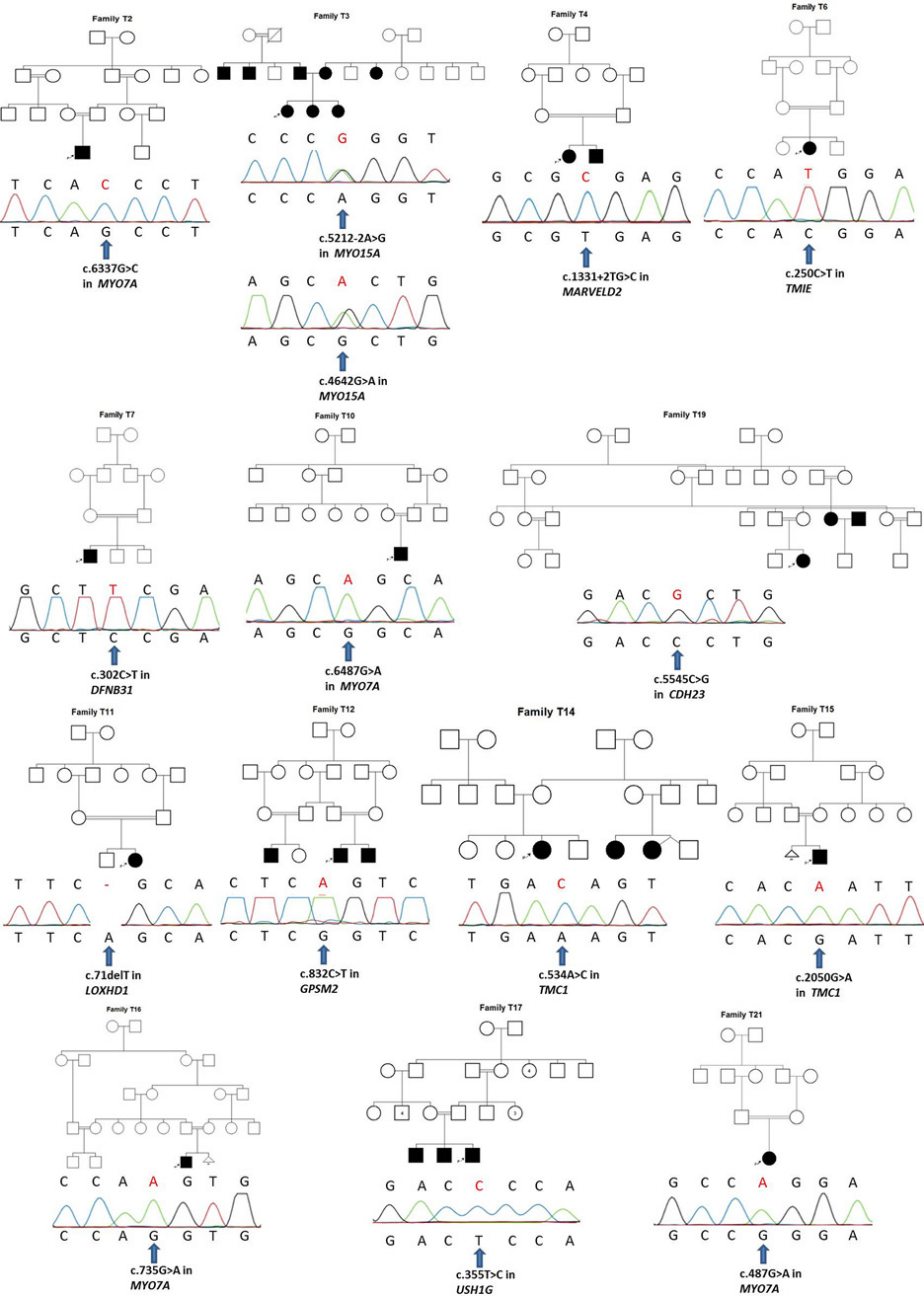
Pedigrees and Sanger sequencing electropherograms demonstrating mutations found in TNGS in the probands.

In this study, using TNGS, molecular diagnosis was established in 14 out of 22 probands who had no mutation in their *GJB2* gene (63.63%). Tables 1, 2 and Fig 1 shows all mutations found in 10 different known ARNSHL genes and their *in silico* prediction analysis. Except two cases, all cases were born to consangiouneous parents.

**Table 2:** Characteristics of the identified variants in the study.

Gene	Transcript ID	cDNA	Protein	Mutation Type	MT	Polyphen2 score/ranking	SIFT	ExAC* ^,^ ^#^ (Overall Allele frequency)	Reference
***MYO7A***	NM_000260	c.487G>A	p.G163R	M	DC	1.000/PD	D	-	Known^24^
***MYO7A***	NM_000260	c.735G>A	-	S	DC	NA	NA	-	Novel
***MYO7A***	NM_000260	c.6337G>C	p.A2113P	M	DC	0.980/PD	D	-	Novel
***MYO7A***	NM_000260	c.6487G>A	p.G2163S	M	DC	1.000/PD	D	0.00003312	Known^23^
***TMC1***	NM_138691	c.534A>C	p.E178D	M	DC	0.992/PD	T	-	Novel
***TMC1***	NM_138691	c.2050G>A	p.D684N	M	DC	0.944/PsD	T	0.00001647	rs563322370
***MYO15A***	NM_016239.3	c.4642G>A	p.A1548T	M	DC	1.000/PD	D	0.00003318	rs201067821
***MYO15A***	NM_016239.3	c.5212-2A>G	-	S	DC	NA	NA	-	rs200760936
***MARVELD2***	NM_001038603.2	c.1331+2T>C	-	S	DC	NA	NA	0.00004119	Known^31^
***TMIE***	NM_147196	c.250C>T	p.R84W	M	DC	1.000/PD	D	0.00001658	Known^34^, rs28942097
***DFNB31***	NM_015404.3	c.302C>T	p.S101F	M	DC	1.000/PD	D	-	Novel
***LOXHD1***	NM_144612	c.71delT	p.L24RfsX74	F	DC	NA	NA	0.00005042	Novel
***GPSM2***	NM_013296	c.832C>T	p.R278X	NS	DC	NA	NA	0.00002473	Novel
***USH1G***	NM_173477.2	c.355T>C	p.S119P	M	DC	0.994/PD	D	-	Novel
***CDH23***	NM_022124.5	c.5545C>G	p.P1849A	M	DC	1.000/PD	D	-	Novel
***GJB2***	NM_004004.5	c.35delG	p.G12VfsX2	F	DC	NA	NA	0.00604	rs80338939
***GJB2***	NM_004004.5	c.299_300delAT	p.H100RfsX14	F	DC	NA	NA	0.00004124	rs111033204
***GJB2***	NM_004004.5	c.333_334delAA	p.K112EfsX2	F	DC	NA	NA	-	Known^16^

* Exome Aggregation Consortium (http://exac.broadinstitude.org)

^#^ The allele frequency in the ExAC database does not contain representative controls for all ethnic groups.

M: Missense, S: Splice site, F: Frameshift, NS: Nonsense, MT: MutationTaster, DC: Disease causing, PD: Probably Damaging, PsD: Possibly Damaging, D: Damaging, T: Tolerated, NA: Not available

## Discussion

In this study, a TNGS panel including known ARNSHL genes has been used to define the genetic etiology in a group of individuals with autosomal recessive NSHL, all recipients of cochlear implants.

In our study, *GJB2* mutations with the direct link to NSHL were found in 7 of 29 probands (24.13%), in line with previous studies, we have confirmed *GJB2* mutations as the principal cause of NSHL in our population. Mutations in *GJB2* have been shown to cause up to 50% of cases with ARNSHL [15]. Although more than 200 different mutations have been identified in this gene, the c.35delG mutation is the most frequent pathogenic variant, possibly accounting for up to 70% of all *GJB2* mutations [15, 16]. In this study, allelic frequency of c.35delG mutation was found to be 78.5% among the patients having *GJB2* mutations (Table 1).

In this study, using the TNGS panel which included 63 known NSHL genes, causative mutations were detected in 63.6% of patients with NSHL (14/22)(Tables 1 and 2). When the patients, identified as having mutations in *GJB2*, were included in the study group, the mutation detection rate increased to 72.4% (21/29). Focusing on familial NSHL cases, Choi et al. (2013) designed a diagnostic pipeline including both prescreening of *GJB2*, *SLC26A4*, *POU3F4* and mitochondrial DNA, and a targeted next generation sequencing panel covering 80 known NSHL genes. In their study included 32 subjects,the total detection rate of 78.1% (25/32) was established [17]. Shearer et al. (2013) used a panel including 89 known deafness genes in 100 probands with presumed genetic NSHL, 39 of these 100 subjects were classified as having autosomal recessive inheritance and molecular defects have been detected in 22 of them (56%) [18]. Vozzi et al. (2014), using a panel including 96 known NSHL genes, found the causative gene variants in four out of 12 families from Italy and Qatar confirming the usefulness of a targeted sequencing approach. In this study, all probands were completely negative for mutations in *GJB2* and *GJB6* genes, as well as for the A1555G mitochondrial mutation at the beginning of TNGS. [1]. More recently, Bademci et al. (2015) reported a different approach to detect deafness-causing variants in a multiethnic cohort with ARNSHL. After excluding mutations in *GJB2*, they performed WES in 160 multiplex families, and sequencing all known NSHL genes, they identified causative mutations in 56% of their cohort [19].

In our study, ExAC database has been used for allelic frequencies of the variants detected. ExAC database may not be ideal for our patients, because it does not contain representative controls from Turkish population. Further studies are needed to exhibit exact allelic frequencies of variants detected in our ethnic group.

*MYO7A* encodes myosin VIIA and has been reported to be associated with Usher syndrome type 1B (USH1B) (MIM 276900), ARNSHL (DFNB2), and ADNSHL (DFNA11) [20–22]. Among previously reported *MYO7A* mutations identified in our study, one (c.487G˃A) had been described in two patients having Usher syndrome by Roux et al. (2006)[23, 24].

*TMC1* has been identified as the responsible gene for both DFNA36 and DFNB7/B11 deafness [25]. To date, more than 30 *TMC1* mutations have been reported to be associated with ARNSHL in families from mostly middle-eastern countries [26]. In two studies conducted in 93 and 86 patients the frequencies of *TMC1* mutations were found to be 4.3% [27] and 8.1% [28] respectively. In our study, 6.89% of patients were carried *TMC1* mutations.

*MYO15A*, encoding the 3530-amino acid myosin XV protein, is one of the common causes of ARNSHL. The frequency of *MYO15A* mutations was found to be 3.3% in a study including 600 consanguineous Pakistani, Indian, and Turkish families [29]. In another study conducted in 140 Iranian deaf families who had no *GJB2* mutations, this frequency was found to be 5.71% (8/140) [30]. In our study, the patient carried *MYO15A* mutations was the only compound heterozygous patient.

Mutations in *MARVELD2*, which encodes tricellulin, and located at the DFNB49 locus, cause ARNSHL [31]. Seven different pathogenic variants of human *MARVELD2* have been identified in patients with moderate to profound hearing loss [31, 32]. Nayak et al. reported that *MARVELD2* mutations were responsible for about 1.5% of NSHL in their cohort of 800 Pakistani families[32]. The splice junction mutation (c.1331+2T>C (IVS4ds+2T-C)) detected in our study has been recurrently reported in Pakistani population [31].

At least 9 mutations of *TMIE*, which encodes 155 amino acid, have been reported to be associated with NSHL [33]. The *TMIE (*c.250C˃T) mutation found in our study had been previously reported by Naz et. al. [34]. Sirmaci et. al. reported that this *TMIE* variant is a founder mutation in southeastern Anatolia region of Turkey. In this previous study of 258 subjects, the frequency of c.250C˃T among families with NSHL throughout Turkey was 3.1% (8/258); while its frequency in southeastern Anatolia was higher (12.2% (6/49))[35].

Mutations in *LOXHD1*, which is responsible for DFNB77, have been extremely rare; there have been only five reports, from six families [36]. In a NSHL patient born to consanguineous parents, we identified a novel homozygous c.71delT (p.Leu24ArgfsTer74) mutation.

One patient was found to carry a novel DFNB31 mutation in our study. Mutations in *DFNB31* coding WHRN protein can cause both ARNSHL and Usher syndrome Type 2 (MIM 607928) [37, 38]. Although at least 19 mutations in *DFNB31* have been reported in the literature, only 2 were presented as being responsible for ARNSHL [37, 39].

*CDH23*, encoding a 3354 amino acid protein, is responsible for both Usher syndrome 1D (USH1D) (MIM 605516) and ARNSHL (DFNB12)[40]. Individuals with USH1D usually have truncating mutations affecting the CDH23 protein, whereas those with DFNB12 usually carry missense mutations in any domain [40]. In our study, a novel homozygous missense mutation (c.5545C˃G) in *CDH23* has been found in a patient with ARNSHL confirming previous findings.

Mutations in *GPSM2* have been previously identified in people with ARNSHL. Although subsequent brain imaging of these neurologically asymptomatic individuals revealed structural brain abnormalities consistent with Chudley-McCullough syndrome (CMS) (MIM 604213), this gene is still accepted as a NSHL gene by http://hereditaryhearingloss.org/ [41, 42]. In our study, a novel homozygous truncating c.832C>T (p.R278X) mutation in this gene was found in a deaf patient. There were no neurological abnormalities other than hearing loss in this patient. Subsequent evaluations have been offered to the family, but they refused any radiological tests.

*USH1G* encodes the protein SANS and is responsible for Usher syndrome Type 1G (MIM 606943). The first cases of ARNSHL caused by mutations in *USH1G* have been presented recently by Maria Oonk et al. (2015), but this gene has not currently been accepted as an ARNSHL gene by http://hereditaryhearingloss.org/ [43]. In our study, a novel homozygous c.355T>C mutation in *USH1G* was found in a NSHL patient born to consanguineous parents. Segregation analysis showed that affected sibling and affected father born to consanguineous parents had this mutation homozygously, while the unaffected mother had the same mutation heterozygously (Family T17 in Fig 1). Neither probands nor other affected family members had abnormality in their ophthalmologic examinations. If we had chosen a custom designed TNGS panel including only known NSHL genes, we wouldn’t have found the *USH1G* mutation in this family. To the best of our knowledge, this family is the second family with NSHL to have a causative mutation in *USH1G*.

As a conclusion, by broadening the spectrum of the gene panels, detection rates will continuously improve making the molecular diagnosis of NSHL easier, cheaper and more available. Molecular genetic diagnosis in the patients with NSHL may also help to improve the development and management of individual treatment strategies such as early speech therapy and cochlear implantation.

## Supporting Information

S1 Table NSHL and SHL genes in TruSight^®^ Exome. (XLSX)
